# *CYP2B6*rs2279343 Is Associated with Improved Survival of Pediatric Rhabdomyosarcoma Treated with Cyclophosphamide

**DOI:** 10.1371/journal.pone.0158890

**Published:** 2016-07-07

**Authors:** Rania M. Labib, Mohamed E. A. Abdelrahim, Enas Elnadi, Reem M. Hesham, Dina Yassin

**Affiliations:** 1 Research Department, Children's Cancer Hospital Egypt 57357, Cairo, Egypt; 2 Clinical Pharmacy, Department, Faculty of Pharmacy, Beni-Suef University, Beni-Suef, Egypt; 3 Pediatric Oncology Department, Beni-Suef University Faculty of medicine, Beni-Suef, Egypt; 4 Pediatric Oncology Department, Children's Cancer Hospital Egypt 57357, Cairo, Egypt; 5 Biochemistry Department, Faculty of Pharmacy, Beni-Suef University, Beni-Suef, Egypt; 6 Molecular Biology Department, Children's Cancer Hospital Egypt 57357, Cairo, Egypt; Johns Hopkins University, UNITED STATES

## Abstract

**Background:**

Rhabdomyosarcoma (RMS) is a small round blue cell malignant tumor, representing 7% of childhood malignancies, and over 50% of all soft tissue sarcomas. Cyclophosphamide (CPA) is a prodrug and is the mainstay of RMS treatment. *CYP2B6* is a highly polymorphic drug metabolizing enzyme involved in CPA bioactivation. The influence of *CYP2B6* single nucleotide polymorphisms (SNPs) on the survival of RMS is still unknown.

**Methods:**

We genotyped *CYP2B6*SNPs rs2279343, rs3745274, and rs3211371 by restriction fragment polymorphism (RFLP) after PCR amplification in a cohort of 73 pediatric RMS patients treated with CPA-based first line treatment. We then analyzed the association between those genotypes and survival outcome of RMS.

**Results:**

The frequencies of *CYP2B6* rs2279343, rs3745274, and rs3211371 were 63%, 45.2%, and 5.5%, respectively. There was no association between rs3745274, rs3211371 genotypes and survival outcomes of RMS. However, the carriers of at least one mutant allele *CYP2B6*rs2279343 had significantly longer event-free survival (*p-value* = 0.03).

**Conclusion:**

Our results demonstrated that *CYP2B6* rs2279343 may predict EFS in RMS patients and warrants future studies to clarify the pharmacogenetics of CPA in pediatrics. If validated, integration of genetic factors with clinical and molecular characteristics could be used for a composite algorithm to better stratify risk prior to treatment.

## Introduction

Rhabdomyosarcoma (RMS) is a small round blue cell tumor, representing 7% of all childhood malignancies [1]. Approximately, 350 new cases of RMS aged less than 19 years old are diagnosed annually in the United States with an incidence of 0.4414 cases per 100,000 children per year [2]. RMS can arise from a variety of anatomic sites and is pathologically subdivided into two distinct subgroups; the alveolar and the embryonal. The embryonal subtype is more frequent and shows better prognosis [3]. Currently, treatment of RMS is based on risk stratification into low, intermediate, and high risk which is determined by both the pre-treatment clinical TNM staging and the surgical grouping [4]. Risk stratification and multimodality care contributed to treatment improvement over the years, where cure rates went from 25% in the early 1970s to 70% over the last 40 years [5]. The combination of vincristine, actinomycin, and cyclophosphamide (VAC) has been used for decades as the standard chemotherapy regimen for treating RMS by the Intergroup Rhabdomyosarcoma Study Group (IRSG). About 35% of RMS can achieve complete response (CR) with chemotherapy alone after initial biopsy or incomplete excision [6].

Cyclophosphamide (CPA) is an oxazaphosphorine alkylating chemotherapeutic agent and is considered the mainstay of RMS treatment. It is a prodrug, which 70–80% of the dose is biotransformed to its active metabolite (4-hydroxycyclophosphamide). Different hepatic cytochromeP450 (CYP450) enzymes have been implicated in CPA activation, including *CYP2B6*, CYP2C9, CYP2C19, and CYP3A4/5. Studies proved that *CYP2B6* is the primary CYP450 responsible for CPA activation [7,8]. *CYP2B6* is highly polymorphic, with several known variant alleles [9,10]. The CYPalleles website (www.cypalleles.ki.se/cyp2b6.htm) currently lists > 100 known single nucleotide polymorphisms (SNPs) in *CYP2B6* in humans with distinct ethnic frequencies [11]. The *CYP2B6* polymorphic variants have been shown to exhibit different metabolic properties than the wild type enzymes [12,13]. With the emerging concepts of personalized medicine, investigators started looking at the role of using common functional *CYP2B6* SNPs to predict response to different *CYP2B6* substrates and to tailor doses for each patient [14]. Nevertheless, their role remains controversial and definitive evidence is lacking, due to sparse, and even contradicting information [15–17].

Understanding the factors affecting CPA metabolism may aid to clarify causes of inter-individual variability in CPA response. The ability to predict treatment failure would help improve CPA dosing regimens. Our hypothesis is that SNPs in *CYP2B6* might affect the functionality of this enzyme in activating CPA, which could be reflected on the response to treatment. Based on such considerations, we conducted this study to investigate the prevalence of 3 *CYP2B6* common functional SNPs rs2279343 (A785G, CYP2B6*4, exon 5, K262R), rs3745274 (G516T, CYP2B6*9, exon 4, Q172H), and rs3211371 (C1459T, *CYP2B*6*5, exon 9, R487C) in our cohort of Egyptian pediatric RMS patients. Our aim was to examine the possible influence of *CYP2B6* polymorphism on the chemotherapy treatment response, and survival outcomes in RMS.

## Materials and Methods

### Patient eligibility and treatment

This retrospective study included 73 histopathologically confirmed RMS patients under the age of 18 years. They were recruited at the Children's Cancer Hospital Egypt (CCHE) from March 2008 until December 2012 and were treated with a combination of vincristine, actinomycin and cyclophosphamide (VAC) as the first line standard clinical treatment for RMS according to IRS-IV protocol. This regimen comprises Vincristine (1.5 mg/m^2^) administered as an IV push (max. 2 mg), actinomycin (0.045mg/kg) as a 5 min intravenous infusion and cyclophosphamide (1.2–2.2 gm/m^2^) infused over 30–60 min, with hydration continued at 3000 ml/m^2^/day followed by a weekly dose of vincristine for 2 weeks. Local control (radiotherapy or surgery) was given in accordance with the treatment protocol. Patients were followed-up till March 2015.

#### Ethics statement

This study was approved by CCHE Institutional Review Board. A written informed consent was obtained from parents/guardians before a patient was enrolled in the study.

#### Blood sampling

Peripheral blood (4 ml) was withdrawn from participants and collected in EDTA vacutainers. Genomic DNA was extracted by the salting out procedure [18]. DNA purity and concentration were measured using Nanoquant ™ spectrophotometer (Infinite M200, TECAN, Switzerland). DNA was kept at –20°C until pharmacogenetic analysis.

### *CYP2B6* genotyping

Genotyping for *CYP2B6* SNPs rs3745274 in exon 4 (G516T), rs2279343 in exon 5 (A785G), and rs3211371 in exon 9 (C1459T) was performed by restriction fragment length polymorphism (RFLP) after polymerase chain reaction (PCR) amplification. All PCR amplifications were carried out in similar standard protocol conditions [10]. Slight modifications were done, for rs3745274 the annealing temperature was 56°C and the extension step was reduced to 40 s while, for rs3211371, a prolonged extension step of 1 min 30 s and the annealing temperature was 65°C. All primers’ sequence for both forward and reverse pairs, restriction enzymes and interpretation of post- RFLP products for the studied SNPs are specified in (Table 1).

**Table 1:** Primers and restriction enzymes used for the analysis of *CYP2B6* polymorphisms.

Exon Amplified	Primer sequence 3'-5'	Length	Primer location	PCR Product size (bp)	Restriction enzyme	Post-Restriction product size (bp)		
						Wild-type	Heterozygous	Homozygous Mutant
Exon 4	Forward:GGTCTGCCCATCTATAAAC	19 bp	Intron 3	526	FastDigest BsrI® (BseNI®, Fermentas)	3 fragments at: 17, 241, and 268 bp	4 fragments at: 17, 241, 268, and 509 bp	2 fragments at: 17, and 509 bp
	Reverse:CTGATTCTTCACATGTCTGCG	21 bp	Intron 4					
Exon 5	Forward:GACAGAAGGATGAGGGAGGAA	21 bp	Intron 4	640	Sty I® (Fermetas)	4 fragments at: 56, 116, 171, and 297 bp	5 fragments at: 56, 116, 171, 297, and 468 bp	3 fragments at: 56, 116, and 468 bp
	Reverse:CTCCCTCTGTCTTTCATTCTGT	22 bp	Intron 5					
Exon 9	Forward:TGAGAATCAGTGGAAGCCATAGA	23 bp	Intron 8	1401	FastDigest BglII® (Fermentas)	1 fragment at: 1401 bp	3 fragments at: 216, 1185, and 1401 bp	2 fragments at: 216, and 1185 bp
	Reverse:AATTTTCGATAATCTCACTCCTGC	24 bp	3'-UT					

#### DNA sequencing

For laboratory validation and substantiation of the protocol and the results capillary Sanger sequencing was used. PCR amplification was performed according to the PCR protocol for the PCR-RFLP assay. Subsequently, the PCR products were purified using the DNA Clean & Concentrator™-25 (DCC™-25) kit, followed by sagner sequencing of both strands using the BigDye® Terminator Cycle Sequencing Kit v.3.1 (Applied Biosystems). The reaction mixture for the sequencing reaction contained 4 μl ABI PRISM Big Dye Terminator (Applied Biosystem), 10 μl water, 2 μl BigDye® Terminator Buffer (Applied Biosystems), 1 μl primer (3.2 pmol) and 3 μl of purified template (40 ng); a total volume of 20 μl. The sequencing conditions were: one cycle at 96°C for 1 min and 25 cycles (96°C for 30 s, 56°C for 5 s, 60°C for 4 min). Forward and reverse sequencing primers were used to sequence both strands of the gene and were the same used for the PCR reactions (see Table 1). The sequence products were purified by gel filtration chromatography using Centri-Sep™ (Thermo Fisher, United States), in order to eliminate excess primers and/or unincorporated dideoxynucleotides (dNTPs). All amplified products were resuspended in 10 μl formamide, and before sequencing analysis, submitted to denaturation at 96°C for 3 min. After being separated on an automated sequencer (ABI PRISM-3130 Genetic Analyzer, Applied Biosystems), DNA sequencing reactions were carried out using a 3730 48-Capillary DNA Analyzer (Applied Biosystems) and MicroAmp Optical 96 Well Plates (Applied Biosystems).

### Clinical evaluation criteria

According to the response evaluation criteria in solid tumors (RECIST) guidelines version 1.0 [19], all patients’ tumor response was evaluated as complete response (CR), partial response (PR), stable disease (StD), or progressive disease (PD).

### Statistical Analysis

The analysis was done using SPSS, version 17 for Windows (IBM Corp., USA), and a *p*-value of ≤ 0.05 was considered a statistically significant result. Descriptive statistic was presented as mean and standard deviation for continuous measures and frequencies and percentages for categorical measures. Tumor objective response outcomes were defined as responders (CR, PR, StD) and non-responders (NR, PD). Correlation between various genotypic variants with treatment outcomes was examined using univariate regression. Association was expressed as odds ratios (OR) with 95% confidence interval (CI). The primary endpoints of the current study were event-free survival (EFS) and overall survival (OS), which were estimated according to Kaplan–Meier method and log-rank test. EFS was defined as the time from the initial diagnosis until the date of disease progression, recurrence, or death due to any cause. The OS was defined as the interval from the initial diagnosis to death or time of the last contact.

## Results

### Patient and tumor baseline characteristics

Table 2 illustrates patients' baseline characteristics. A total of 73 pediatric RMS patients were enrolled receiving CPA as part of their clinical treatment protocol for RMS. The study population had a median age of 4.54 years (range, 0.07 to 17.4 years); the mean age was 5.4 ± 4.2 years and included 46 male and 27 female patients. The mean body weight, height, and surface area were 25.1 ± 17.6 kg, 115.7 ± 27.8 cm and 0.86 ± 0.39 m^2^, respectively. Regional nodal involvement (N1) occurred in 30 (45.2%), and there was distant metastasis (M1) in 19 patients (26%).

**Table 2:** Baseline characteristics of the study cohort (n = 73).

***Variables***	***(Mean ± SD)***	
Age (years)	5.4 ± 4.2	
Body surface area (m^2^)	0.86 ± 0.39	
Weight (kg)	25.1 ± 17.6	
Height (cm)	115.7 ± 27.8	
***Variables***	***Category***	***Number (%)***
Gender	Male	46 (63%)
	Female	27 (37%)
Age category	≤ 1 years	10 (13.7%)
	1–10 years	51 (69.9%)
	≥10 years	12 (16.4%)
Site	Favourable	25 (34.2%)
	Unfavourable	48 (65.8%)
Histopathology	Alveolar	19(26%)
	Embryonal	54 (73.9%)
Tumor group	I	10 (13.7%)
	II	3 (4.1%)
	III	41(56.2%)
	IV	19 (26%)
Clinical Stage	I	25 (34.2%)
	II	9 (12.3%)
	III	20 (27.4%)
	IV	19 (26%)
Tumor size	≤ 5 cm	32 (43.8%)
	> 5 cm	37 (50.7%)
	Unknown	4 (5.5%)
Lymph nodes	N_0_	33 (45.2%)
	N_1_	30 (41.1%)
	unknown	10 (13.7%)
Risk	Low	12 (16.4%)
	Intermediate	42 (57.5%)
	High	19 (26%)

### Genotyping and allele frequency

**Fig 1:**
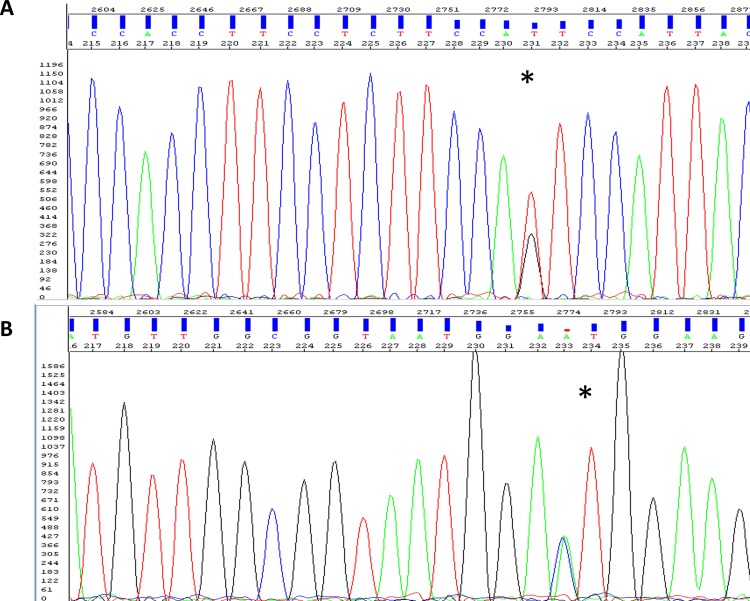
Electropherograms of *CYP2B6*rs2279343 mutation in forward and reverse sequences. The patient is a heterozygous variant carrier. Asterisks denote the mutated base. **A**: Electropherogram of the forward. **B**: Electropherogram of the reverse.

The distributions of the genotypes are shown in Table 3. In our cohort, 63% carried rs2279343 (50.7% as heterozygous and 12.3% as mutant homozygous), 45.2% had rs3745274 (37% as heterozygous and 8.2% as mutant homozygous) and 5.5% had rs3211371 mutations (4.1% as heterozygous and 1.4% as mutant homozygous). The frequency of having the minor mutant allele whether homozygous or heterozygous was 0.38, 0.27 and 0.03 for rs2279343, rs3745274, and rs3211371, respectively. PCR-RFLP was validated by sequencing as shown in (Fig 1).

**Table 3:** Genotype frequencies of the explored genetic polymorphisms (n = 73).

rs number	Nucleotide, (AA)	Wild genotype (number, %)	Heterozygous genotype (number, %)	Mutant genotype (number, %)	Minor mutant allele frequency
rs2279343	A785G, (K262R)	AA (27, 37%)	AG (37, 50.7%)	GG (9, 12.3%)	0.38 (G)
rs3745274	G516T, (Q172H)	GG (40, 54.8%)	GT (27, 37%)	TT (6, 8.2%)	0.27 (T)
rs3211371	C1459T, (R487C)	CC (69, 94.5%)	CT (3, 4.1%)	TT (1, 1.4%)	0.03 (T)

rs number = Reference SNP number, AA = amino acid change

### Response and survival function

**Fig 2:**
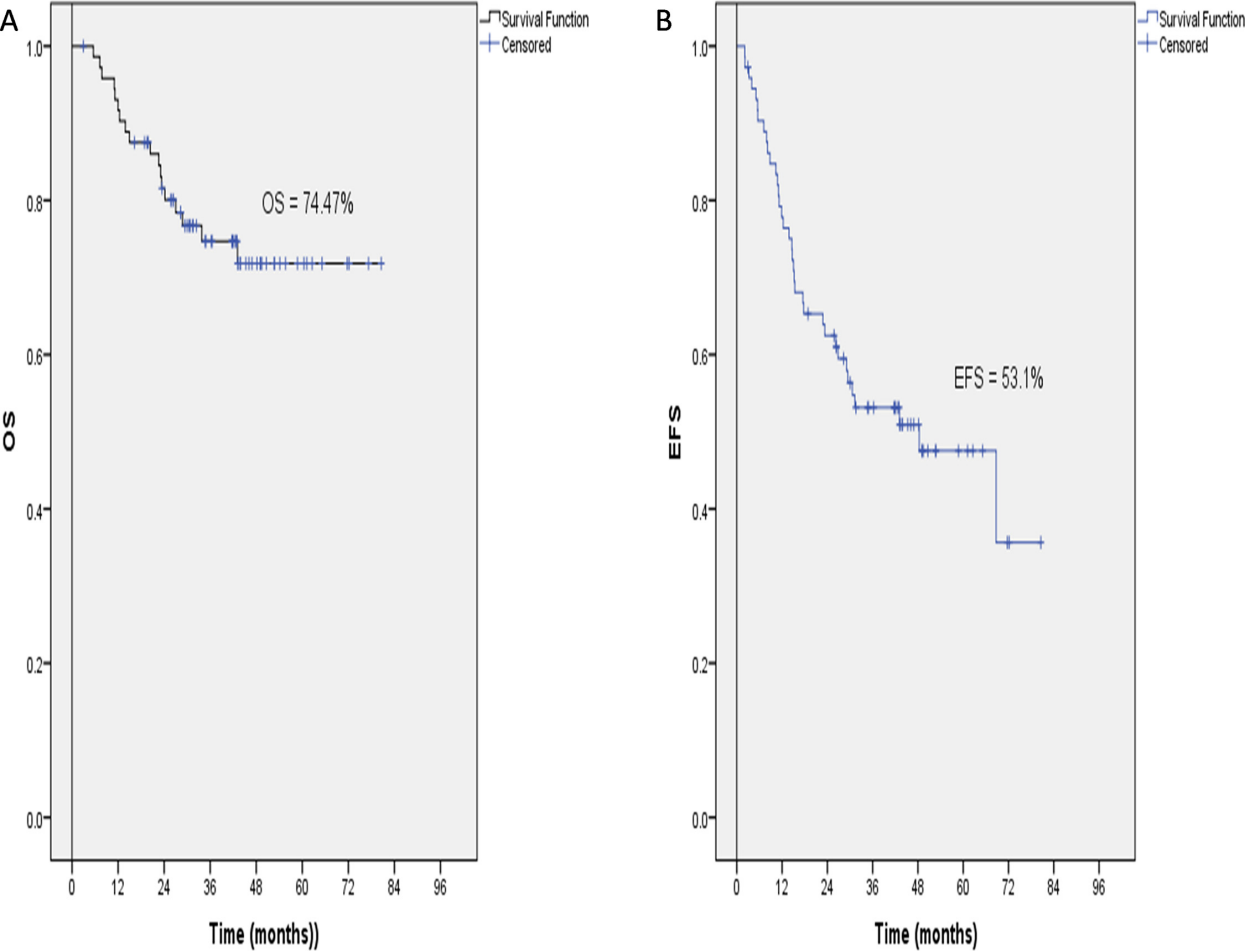
Kaplan-Meier illustrating OS and EFS of the study cohort. (a) 3-Years OS of the study cohort.3-years OS is 74.47 ± 51%, 95%CI is (56.26–70.06). (b) 3-Years EFS of the study cohort. 3 years EFS = 53.1 ± 6. (95%CI is 38.47–54.2)

The end points used for clinical outcome measures in this study were clinical response, EFS, and OS. The mean follow-up period was 43.9 ± 14.7 months. The mean OS was 63.73 ± 3.4 months (95% CI: 57–70.4). The mean EFS was 48.3 ± 12.5 months (95% CI: 23.78–72.92). At a median study follow-up of 43 months (range, 16.3–80.6 months), the EFS and OS were 53.1% and 74.47%, respectively (Fig 2). There were 39/73 (53.4%) responders to first line treatment.

#### Association of *CYP2B6* genotypes with objective clinical response, OS, and EFS

**Fig 3:**
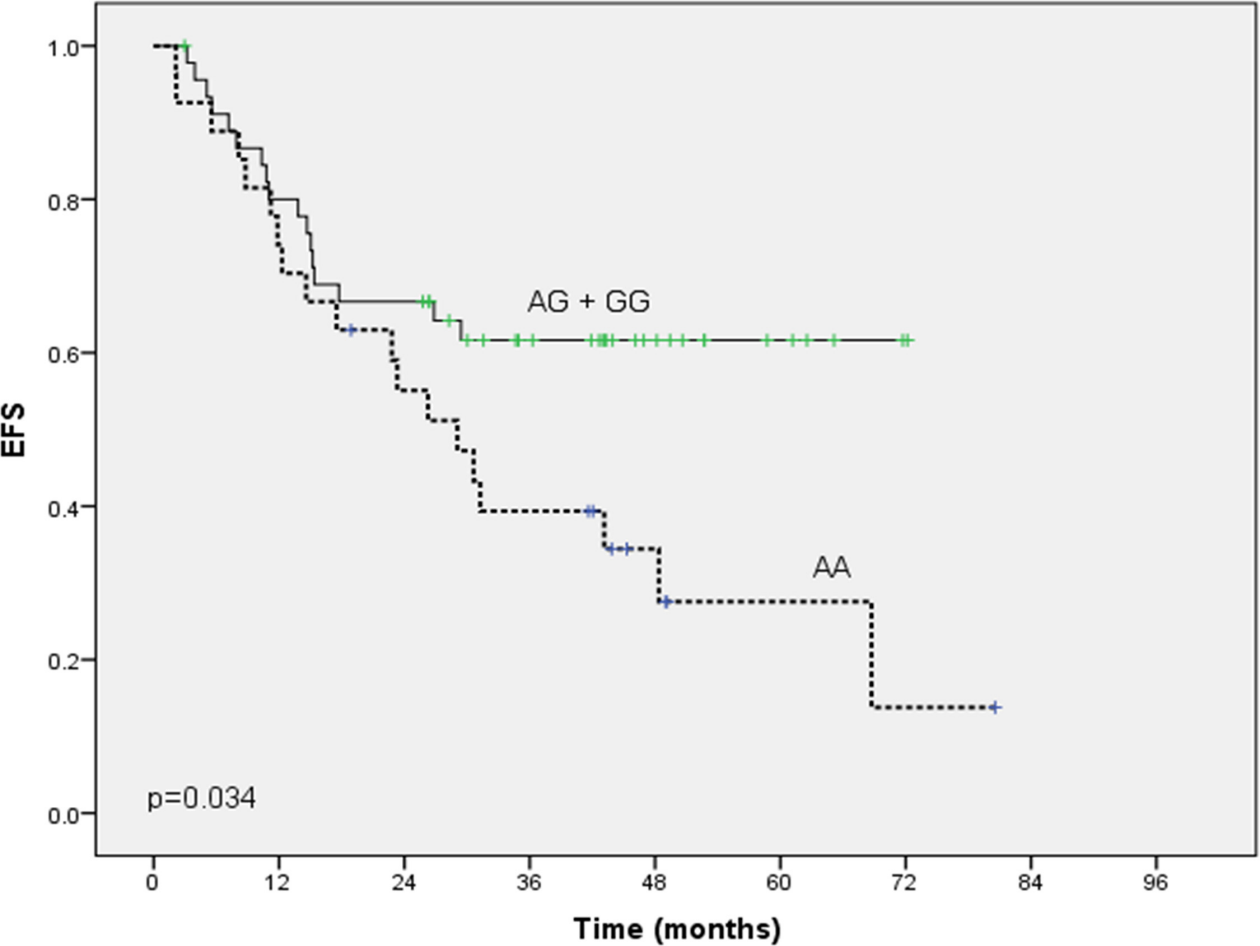
Kaplan-Meier analysis of EFS according to *CYP2B6* rs2279343. Kaplan-Meier curves are shown for a dominant genetic model of inheritance (p-value = 0.034). The log-rank test was used to calculate P-values.

The survival data showed that 3-years EFS after VAC chemotherapy was 53.1%. Table 4 summarizes the association of *CYP2B6* SNPs with the objective response and the survival outcome. Carriers of at least one mutant allele rs2279343 (n = 46/73) when compared to wild-type homozygotes, showed a significant association with the clinical objective response (p-value = 0.01), and a significantly longer EFS (p-value = 0.03) (Fig 3), but there was no discernable effect on the OS (p-value = 0.48). On the other hand, there was no observed association between neither rs3745274 nor rs3211371 with any of the study end points.

**Table 4:** *CYP2B6* SNPs correlated with objective response and survival outcome (crude analysis).

Genetic Model	Genotype	Objective Response				OS			EFS		
		Responders N (%)	Non-Responders N (%)	OR (95% CI)	P-value	Mean (months)	95% CI	P-value	Mean ± SD (months)	95% CI	P-value
rs2279343 Dominant	AA	9 (23.1%)	18 (52.9%)	1	**0.008**	60.2 ± 5.9	48.6–71.7	0.48	35.9 ± 5.5	25.2–46.6	**0.03**
	AG / GG	30 (76.9%)	16 (47.1)	**3.75 (1.37–10.24)**		59.5 ± 3.6	52.5–66.5		49.4 ± 4.4	38.5–54.2	
rs3745274 Dominant	GG	20 (51.3%)	20 (58.8%)	1(ref)	0.52	63 ± 4.7	53.6–72.3	0.85	43.6 *± 5*.*2*	33.4–53.7	0.61
	GT / TT	19 (48.7%)	14 (41.2%)	1.36(0.54–3.43)		58.4 ± 4.2	50.6–67		45.2 *± 5*.*2*	35–55.4	
rs3211371 Dominant	CC	36 (92.3%)	33 (97.1%)	1(ref)	0.36	Mean was not computed as all cases were censored		0.27	45.3 *± 4*.*1*	37.2–53.3	0.33
	CT / TT	3 (7.7%)	1 (2.9%)	2.75(0.27–27.76)					42.3 *± 7*.*2*	28.3–56.4	

The dominant model is comparing the wild type versus having 1 or 2 mutant alleles. rs number = Reference SNP number, OS = Overall-survival, EFS = Event-free survival. Association between study genotypes and the clinical objective response were tested for statistical significance with the Chi-square test. Correlation with the OS and EFS were tested by log-rank test.

## Discussion

CPA is an anticancer prodrug, which in order to exert its antitumor activity requires bioactivation to the active alkylating metabolite, 4-OH-CPA [20]. There is a significant heterogeneity in CPA treatment response. *CYP2B6* was identified as the major CPA 4-hydroxylase catalyzing the metabolism of CPA [7,8]https://en.wikipedia.org/wiki/Cyclophosphamide-cite_note-3. Strategies to identify patients at risk of treatment failure and those who will require further dose adjustment might help in decisions regarding treatment doses, intensifying chemotherapy doses or length of treatment [21]. Many studies investigated the impact of *CYP2B6* on the clinical outcome of different substrates (e.g bupropion, efavirenz, bevacizumab) and concluded that *CYP2B6* effect is substrate specific [14–17]. A previous study has found a significantly higher incidence of relapse and graft failure in 66 pediatric patients receiving a busulfan-based conditioning regimen carrying a homozygous reduced functional *CYP2B6* allele compared with carriers of at least one wild allele [22]. On the other hand, some studies did not show any effect of *CYP2B6* polymorphisms on CPA treatment outcome [23,24].

Altogether in vivo and in vitro studies have not reached a conclusive evidence to support the role of different *CYP2B6* polymorphisms in predicting CPA effect. Helsby and Tingle attributed this inconsistency of data to different study size design as well as lack of consistency in allele definition and genotype information among studies [25]. Yet, our study is one of few pharmacogenetic studies carried out on pediatric population aiming to investigate the influence of *CYP2B6* variants on CPA treatment outcome.

Based on this, rather than choosing a “genome association study approach” with complex data analysis, our study design was based on a “candidate SNP approach” to examine particular SNPs in CPA activation pathway that are thought to influence response to treatment. We have selected 3 common functional SNPs (rs3745274, rs2279343, and rs3211371) found in *CYP2B6* exons 4, 5, and 9, respectively. They were carefully selected based on evidence that they are common in Caucasians [10], they cause changes in *CYP2B6* coding that would affect *CYP2B6* enzyme expression or activity and are associated with a change in CPA activation and metabolism [24,26]. Our hypothesis is that the pharmacological activity of CPA could be altered by the functionality of *CYP2B6* which might be reflected on the treatment response. Thus, interpatient variability in CPA response may be due in part to altered CPA metabolism by the *CYP2B6* polymorphic variants. In light of these considerations, the aim of this study was to analyze the frequencies of allelic variants of *CYP2B6* in pediatric Egyptian RMS patients. Furthermore, to determine if they influence treatment efficacy and survival outcome and hence, can be used as a prognostic biomarker for response. A total of 73 children with RMS were recruited and genotyping of the selected SNPs were analyzed with all the clinical data and outcome.

*CYP2B6* is known to have inter-ethnic variability, resulting in a high genetic diversity among different populations as shown in Table 5. In our cohort, the percentage of patients carrying at least one copy of *CY2B6*rs2279343, rs3745274, and rs3211371 were 63%, 45.2%, and 5.5%, respectively.

**Table 5:** Distribution of the study *CYP2B6* SNPs in different ethnic population.

Population	rs2279343	rs3745274	rs3211371	Ref
In our cohort	63%	45.2%	5.5%	Our results
Caucasians	33%	29%	--	[10]
African Americans	26%	41.7%	2.1	[27]
European Americans	17.4%	33.3%	36.2%	
Japanese	13.5%	32.3%	0	
Han Chinese	11.4%	24%	0	
Koreans	18.9%	11.5%	0	
Italians	2.5%	0	--	[28]

The main finding in our study was that in RMS patients treated with VAC as the first line of treatment, there was an association between *CYP2B6*rs2279343 (carrying G mutant allele) and response to CPA-based treatment. Patients who carried at least one mutant allele *CYP2B6*rs2279343 (n = 46/73), had better objective clinical response compared to the homozygous wild allele carriers (p-value = 0.01). Also, our results demonstrate that this group of patients who carried at least one mutant allele in *CYP2B6*rs2279343 (G allele), had a significant longer EFS (p-value = 0.034) but not OS (p-value = 0.48) compared to those carrying the homozygous wild allele (A allele). Our results have provided evidence for the involvement of *CYP2B6*rs2279343 in the response of RMS patients to CPA-based treatment. This is in accordance with a previous study that identified a strong functional impact of rs2279343 on enhancing the catalytic activation of CPA into 4-OH-CPA and a tendency to reduce protein expression [8]. In contrast to several studies which associated variants including *CYP2B6*rs2279343 with lower CPA hydroxylation or with poor response [17,29]. Raccor et al. found that *CYP2B6*rs2279343 genotype caused 50% reduction in 4-OH-CPA formation yet the sample size was too small to draw a reliable conclusion. In addition, they noted that carrying rs2279343 alone or rs3745274 alone displayed a reduction in intrinsic CPA clearance compared with the wild type [30]. In this context, our hypothesis generated in this study is that patients carrying *CYP2B6*rs2279343 (heterozygous or mutant homonozygous genotypes) have a predicted rapid metabolic phenotype; consequently, maintaining an increased CPA activation to 4-OH-CPA, while patients with the wild-genotype will have a higher ratio of CPA to 4-OH-CPA in plasma. Thus, carriers of at least one mutant allele *CYP2B6*rs2279343 have a higher success rate for RMS therapy with CPA.

In summary, our results provide a picture of the role of *CYP2B6* polymorphisms in RMS. Our data suggests that pretreatment evaluation of *CYP2B6*rs2279343 may explain some inter-individual differences in treatment response. However, this should be replicated in a larger cohort and more studies are warranted to clarify the pharmacogenetics of CPA in pediatrics. If validated, integration of genetic factors with clinical and molecular characteristics could be used for a composite algorithm to better stratify risk prior to treatment. Moreover, this may facilitate clinical decision, improve CPA treatment, and hence improve the clinical outcome. Nevertheless, one limitation is that our study did not correlate the genetic variation with serum drug metabolite levels. The pharmacokinetic modeling of CPA activation will give a more clear understanding of the inter-individual variation in drug response and how to stratify RMS patients based on their genetic makeup.

## Abbreviations

RMS: Rhabdomyosarcoma
CPA: Cyclophosphamide
CYP2B6: Cytochrome P-450 *CYP2B6* enzyme
SNPs: Single nucleotide polymorphisms
PCR-RFLP: Polymerase Chain Reaction- Restriction Fragment Length Polymorphism
OS: Overall Survival
EFS: Event-Free Survival
